# Medical College and Hospital Notes

**Published:** 1897-11

**Authors:** 


### Medical College and Hospital Notes.

The Medical Department of Niagara University, during the present school session, bids fair to have a course in bacteriology second to none in the state. Dr. William G. Bissell, bacteriologist to the health department of Buffalo, will have charge of the course, which will consist mostly of practical laboratory work. The course is intended to extend over a period of sixteen weeks, eighty hours of which time will be devoted entirely to practical laboratory work, with an additional eighteen hours of lectures. The bacteriological laboratory has been recently equipped with the most modern apparatus, including incubator, autoclave, sterilizers, microscopes, and the like, besides every facility for animal inoculation work. Dr. Bissell will be assisted during the course by Dr. U. B. Stein, who has but recently returned from Europe, where he had instruction in two of the most prominent laboratories ; also by Mr. Baker and Mr. Ryan, former students of Dr. Bissell.

A cordial invitation is extended to members of the medical profession to attend the lectures, the opening one of which will be given Wednesday, November 3d, at 5 P. M., at the College building, 203 Ellicott street. The remaining lectures will be given on Friday of each following week at the same hour.

---

The Buffalo Hospital of the Sisters of Charity training school for nurses held its first formal graduating exercises on Tuesday evening, October 5, 1897. Dr. Henry D. Ingraham presided and delivered an introductory address, in which he said the training school was organised about eight years ago and was the first instituted in a sisters' of charity hospital in the United States. Vocal music followed, given by Miss Cronyn, Miss Carbone, Dr. Mooney and ex-judge Lewis. The Rev. Francis Lobdell, rector of Trinity church, delivered the address to the graduates, in which he took occasion to refer to the importance of nursing as a calling and of ample preparation for the work ; especially, he said, should a nurse be refined, educated and faithful. Dr. Mooney sung a tenor solo and then Dr. Ingraham presented the diplomas in the following terms : "In consideration of your qualifications I present you, on behalf of the sisters and the hospital staff, these diplomas as a certificate of your ability. You thus become graduated nurses with all the honor, as well as all the responsibility, which the title implies. You will look for the ideal and endeavor to put it into the real, that you may become perfect women as well as perfect nurses."

The graduates are: Antoinette L. Weber, Hamburg; Mary C. Hoffman, Oneida; Anna E. Dunn, Owego; Helen S. Alt, Erie, Pa.; Rose E. Gill, Belfast; Ellen M. Ryan, Buffalo; Mary H. Kennedy, Buffalo. Following another quartette by Miss Cronyn, Miss Carbone, Dr. Mooney and Mr. Lewis, the Rt. Rev. James E. Quigley, D. D., Bishop of Buffalo, presented medals to the graduates and addressed them in a few well-chosen words.

---

Dr. Herbert U. Williams, professor of pathology and bacteriology at the Buffalo University Medical College, announces a course of four lectures on bacteriology, to be delivered upon the occasion of the opening of the new bacteriological laboratories at the college, to be illustrated in part by lantern pictures. The first lecture was given Monday, October 25, 1897, at 5 o'clock P. M., in alumni hall. Special invitations have been issued to the medical profession to attend the course, the receipt of one of which the Journal is pleased to acknowledge.

---

The Philadelphia Polyclinic and College for Graduates in Medicine will hold its fifth special week in ophthalmology commencing November 15, 1897, which will be devoted chiefly to ophthalmic operations. It will include lectures, demonstrations and clinics at the Polyclinic and other hospitals, with conferences at which the latest suggestions and special technique of the various operations will be discussed.

When not otherwise stated the work will be at the Polyclinic Hospital, Lombard street, between 18th and 19th streets.

The fee for the full course, including all clinical work, is $15 ; or for the special lectures, demonstrations and conferences (shown in italics on the roster) without the regular clinics, $5.

ROSTER.

Monday, November 15th.—9 A. M., *Retinoscopy*, Dr. Thorington; 12 M., The operative treatment of detachment of the retina, Dr. Wallace; 2 P. M., Clinic at Wills Eye Hospital—Cataract operations, Dr. Risley; 4 P. M., Clinical work, Dr. Schneideman, and clinical demonstrations and operations, at Blockley Hospital, Dr. De Schweinitz; 8 P. M., *Conference on operative procedures for glaucoma.*

Tuesday, November 16th.—9 A. M., *Removal of the lens for high myopia*, Dr. Jackson; 11 A. M., Clinical demonstrations at the Children's Hospital, Dr. Thomson; 12 M., *The operation of tenotomy*, Dr. Hansell; 2 P. M., Clinic at Wills Hospital—Cataract extraction, Dr. Jackson; 4 P. M., Operative technique, illustrated on animals' eyes, Dr. De Schweinitz and Dr. Veasey; 8 P. M., *Operations on the eye and lids.*

Wednesday, November 17th.—9 A. M., *The technique of cataract operations*, Dr. Carpenter; 12 M., *Use of the Röntgen Rays in ophthalmic surgery*, Dr. Sweet; 2 P. M., *Clinic at Wills Hospital*, Secondary glaucoma, iridectomy, Dr. Risley; 4 P. M., Clinical demonstrations, Dr. Schneideman; operations at the Blockley Hospital, Dr. De Schweinitz; 8 P. M., *Operations on the ocular muscles.*

Thursday, November 18th.—9 A. M., *Retinoscopy*, Dr. Thorington; 12 M., Clinical work, Dr. Hansell; 2 P. M., Clinics at Wills Hospital—Needle operations for cataract, Dr. Jackson; 4 P. M., *Operations on the ocular muscles for abnormalities of ocular balance*, Dr. Risley; 7 P. M., *Operative interference in orbital disease.*

Friday, November 19th.—9 A. M., *The refractive changes after corneal sections*, Dr. Jackson; 11 A. M., Clinical demonstration at Children's hospital, Dr. Thomson; 12 M., *Ocular asepsis and antiseptics*, Dr. Reber; 1 P. M., Clinic at Jefferson Hospital, operations, Dr. Hansell; 2 P. M., Clinics at Wills Hospital, strabismus, Dr. Risley; 4 P. M., *Opening the lachrymal passages*, Dr. Schneideman; 8 P. M., *The treatment of lachrymal obstruction.*

Saturday, November 20th.—9 A. M., *Antisepsis and sepsis in operations upon the eye*, Dr. Carpenter; 12 M., Traumatisms of the cornea, Dr. Haydon; 2 P. M., Clinics at Wills Hospital, operations, Dr. Jackson; 4 P. M., Treatment of incised wounds of cornea and iris-prolapse, Dr. De Schweinitz.

